# Factor Associated With Teacher Satisfaction and Online Teaching Effectiveness Under Adversity Situations: A Case of Vietnamese Teachers During COVID-19

**DOI:** 10.1177/00220574211039483

**Published:** 2021-11-06

**Authors:** Phuong-Tam Pham, Thanh-Thao Thi Phan, Yen-Chi Nguyen, Anh-Duc Hoang

**Affiliations:** 1 95400 Can Tho University, Can Tho, Vietnam; 2 Thanh Do University, Hanoi, Vietnam; 3 575875 EdLab Asia Educational Research and Development Centre, Hanoi, Vietnam; 4 School of Business and Management, RMIT University, Hanoi, Vietnam

**Keywords:** teacher performance, teacher satisfaction, Vietnam, COVID-19, SDG4

## Abstract

How teachers perform and react to the world-wide pandemic and how the epidemic affects an education system may also be used as new conditions to consider the way to enhance SDG4 in developing countries. Regarding that concern, this study investigated 294 teachers’ perspective on their teaching effectiveness and satisfaction during COVID-19. The findings underlined the significant roles of support from various stakeholders, school readiness toward digital transformation, and teachers’ anxiety over teacher satisfaction. Notably, teachers’ newly absorbed technological and pedagogical skills do elevate their teaching effectiveness but do not lead to higher satisfaction during the pandemic.

There is no boundary that can stop the impact of COVID-19 across countries and industries. As a country which started dealing with the COVID-19 very soon, Vietnam has applied a school closure policy for schools all over the nation since February 4, 2020. The Vietnamese education system has to face a trilemma of ensuring the safety, learning progress, and proper living standard for teachers. On the one hand, the threats of SAR-COV-2 virus over students and teachers were limited. On the other hand, there are massive changes in teaching and learning habits as online learning was not a popular solution in the country (T. Tran, et al., 2020). In addition, more than one million Vietnamese teachers have to upgrade themselves to master new technologies while the concerns about the future always exist in their mind.

Expanded from Wuhan, China, since early January 2020, the COVID-19 has been labeled with different levels of risk by various governments (Callaway, 2020). Some governments decided to adopt a social distancing policy in early February (Brahma et al., 2020), while other cabinets did not tighten their preventive regulations until early April (University of Oxford, 2020). Empirical evidence from prior coronavirus epidemics reported low levels of transmission in schools. However, by March 18, 2020, the school closure policy has been applied in 107 countries (Viner et al., 2020). Till early May, more than 1,268 million students, which are about 72% of total learners across 177 countries and territories, were affected by the COVID-19 (UNESCO, 2020). To minimize the negative impact of school closure, universities and educational institutions over the globe established different platforms, resources, and guidelines to fulfill the gap of teacher competency (Cambridge University, 2020; VPAL, 2020) and elevate learning and teaching practices (MHA, 2020). Notwithstanding, the sudden digital transformation still did not adapt to the teaching and learning demand, as well as caused new educational inequality (Hodges et al., 2020). Such unforeseen changes lead to adverse effects over students, parents, and teachers (Hoang, 2020; Hoang et al., 2020; T. Tran, et al., 2020), not to mention the other damages to economics and society (Bin Nafisah et al., 2018; Rashid et al., 2015).

As a very close neighboring country with 1.435 km of shared land borders with China, Vietnam alerted the high risk of COVID-19 since very early (La et al., 2020). Besides the debate on the effectiveness of national school closure policy (T. Tran, A.-D. Hoang, Y. C. Nguyen, L.-C. Nguyen et al., 2020), there are also arguments on tuition that parents have to pay during school closure (Nguyen et al., 2020). While teachers in public schools were not affected by the tuition contest, teachers in private schools struggled, especially kindergarten teachers, whose grade levels are not appropriate for online classes. Specifically, among nearly 42,000 teachers had postponed working contracts and no salary, 29,700 of those were kindergarten teachers (Thanh, 2020). On March 3, 2020, a group of more than 150 private education institutions proposed a letter to the Vietnamese government, asking for support on policy, regulations, and taxation. Accordingly, about 70% of private education institutions will go bankrupt in the next 3 months as their cash flows were disrupted (Nguyen, 2020). Tackling the cost and damage of COVID-19 is a crucial mission of scientist, especially researchers in developing countries like Vietnam (Vuong, 2018). Regardless of the sources and the level of issues, these difficulties seriously challenge teachers’ motivation and commitment to the teaching profession (Canrinus et al., 2012). Thus, sustaining teachers’ mental health during the pandemic is also very important.

Teaching effectiveness and teacher satisfaction are the main concerns of educational leaders in most countries and territories (Mulford, 2003; OECD, 2005). However, there is a lack of studies on teacher satisfaction and teaching effectiveness under the circumstance of school closure and social distancing policies due to pandemic. Thus, besides responding to the call of researching to prevent and minimize the impact of COVID-19 (Elseviers, 2020), this study also extended literature on teacher satisfaction and effectiveness, with a focus on the chaotic situation of a global crisis. This empirical evidence can contribute to elevating the way teachers overcome future adversity situations. Concerning the influence of stress, perceived support, school readiness, and teachers’ newly absorbed skills over their satisfaction and teaching effectiveness, this study focused on those following research questions:

1. How do teachers’ perceptions on the impact of COVID-19 affect their satisfaction and online teaching effectiveness?
2. How do teachers’ perceived support affect their satisfaction and online teaching effectiveness?
3. How does school readiness toward online learning affect teacher satisfactions and online teaching effectiveness?
4. How do teachers’ newly absorbed knowledge and skills affect their satisfaction and online teaching effectiveness?

## Literature Review

### Teacher Satisfaction

Teacher satisfaction is crucial to the operational excellence of any educational institution. Satisfaction in the teaching profession is quite different from other occupations due to its nature mechanism (Jorde-Bloom, 1986), antecedents, and outcomes (Skaalvik & Skaalvik, 2011). As life-long learners, teachers always seek new opportunities to develop themselves and raise their standards continuously (Little, 1995). Thus, maintaining intrinsic and extrinsic motivations is an essential concern, regarding the need for sustainable education quality (Hoang et al., 2020b; Pearson & Moomaw, 2005). Scholars have noticed that there are two broad measures or aspects of peoples' career satisfaction instead of a classical concept of a simple continuum or single measure (Holdaway, 1978; Nias, 1981). On the one hand, teachers are most satisfied by matters intrinsic to the role of teaching. On the other hand, teachers are dissatisfied with extrinsic issues to the task of teaching and working, such as the broader domain of society, governments, and the employing body (Dinham & Scott, 1996).

Recently, newly proposed clustering approaches for teacher job satisfaction follow the initial research of Hofstede (1980) on six dimensions of cultural values, which include an aspect of collective versus individual behavior (Klassen et al., 2010). In particular, different cultures also lead to various contributions to the notion of teacher job satisfaction. Teachers from collective cultures like East Asian countries often have higher job commitment due to higher eagerness to prolong to directorial settlement (Kirkman & Shapiro, 2001). Even for Chinese teachers who are working in Western countries, the teachers who tribute the long-established value of following leaders also experience pressure adversely compared to their countryman who pared down that traditional belief (Xie et al., 2008). Disregarding the identical and cultural aspects, Leithwood and Sun (2012) stated that leadership could influence both the collective behavior and individual behavior of teachers. Caprara et al. (2003) confirmed that teachers' performance does enrich teacher job satisfaction in both collectivistic and individualistic cultures. However, Muhammad Arifin (2015) made an important point that despite the significant influence of motivation over teacher job satisfaction, its impact on teacher efficiency still needs more investigation.

Factors associated with teachers’ satisfaction can be categorized by the origin of the problem (internal-caused or external-caused) (Thibodeaux, 2014) or the level of challenge (Klassen et al., 2010). In particular, teachers themselves encounter adversity situations that generate conflicts and stress (Cooper & Travers, 2012; Gold & Roth, 2013). Besides teacher’s perceived unfairness and sadness, different stakeholders such as students, colleagues, school managers, school administrators (Sergiovanni, 1967), and media (Hargreaves & Fullan, 1998) can also influence teacher job satisfaction. Regarding the hierarchy of factors associated with teacher satisfaction, Dinham and Scott (1998) proposed an eight-factor model to capture the notion of teacher satisfaction over three domains which are as follows: core teaching activities, school-related factors, and society-related factors. Day et al. (2007) and Dörnyei and Ushioda (2013) both confirmed that indicators related to the core teaching profession as the most critical factor which maintain a high level of teacher satisfaction. In contrast, they also stated that it is not easy to make teachers happy with school leadership, working culture, organizational structure, decision-making process, or school reputation. Admit that challenges, any action aiming to enhance teacher satisfaction by tackling school-level issues, are often acknowledged by teachers (Leithwood & Sun, 2012; Nguni et al., 2006).

Regardless of the sources and the level of issues, these difficulties seriously challenge teachers’ motivation and commitment to the teaching profession (Canrinus et al., 2012). Accordingly, we suggested four hypotheses to examine the influence of teacher perceptions, the support they received, the readiness of their school toward online learning during the pandemic, and their newly absorbed competencies, over teacher satisfaction during COVID-19.

Hypothesis *1a:* Stress feeling of COVID 19 has a negative impact on teacher satisfaction.

Hypothesis *2a:* Teacher perceived supports have a positive impact on teacher satisfaction.

Hypothesis *3a:* School readiness toward online learning has a positive impact on teacher satisfaction.

Hypothesis *4a:* Newly absorbed knowledge and skills during the pandemic period have a positive impact on teacher satisfaction.

### Online Teaching Effectiveness

#### Teaching Effectiveness

Teaching is one of the most stressful occupations (Johnson et al., 2005), in which teachers have to maintain high levels of performance regardless of their condition (Chaplain, 1995). Both the early-stage teachers to experienced teachers, from under-graduated students to professors, have to face the stressful nature of teaching (Chaplain, 2008; Kyriacou, 1987). There are three factors of performance, including task performance, citizenship behavior, and counterproductive behavior (Colquitt et al., 2011). Teacher performance, therefore, is the demonstration of their impact on students learning and can be measured through student achievement, pedagogical practice observation, school, or student survey results (Lieberman & Miller, 1984). Teacher effectiveness is the aggregated impact of teacher behaviors on student learning (Chi et al., 2013; Seidel & Shavelson, 2007). Marsh and Bailey (1991) proposed the multi-dimensional approach to measure teacher effectiveness, including learning value, teaching enthusiasm, clear expression, group interaction, the harmonious relationship between teachers and students, tr context, evaluation methods, extracurricular assignments, and learning difficulty. Notwithstanding, teacher effectiveness influences student achievement the most, in comparison with other determinants like class size, in-class composition, or previous student achievement (Darling-Hammond & Youngs, 2002; Staiger & Rockoff, 2010).

Teachers’ psychological characteristics have long been hypothesized to contribute to teaching effectiveness (Barr, 1952). Therefore, the state of well-being is necessary for effective teaching performance. Calabrese (1987) featured that teachers with higher teaching effectiveness and engagement are the ones who have a lower level of stress. Overall, factors related to the organizational culture, such as working pressure, learning cultural, and interpersonal issues with students or colleagues, can lead to a higher level of teacher stress (Antoniou et al., 2006). For instance, the most common sources of stress for almost teachers are student behavior and workload (Boyle et al., 1995). Regarding the internal factors that affect teacher performance, Maddux (1995) stated that teacher self-efficacy is the most crucial toward teacher performance. In particular, teachers’ personality, perception, emotion, and cognitive determine the way they develop their learning capabilities, as well as social interaction toward better performance. In some specific circumstances, physical and mental support is needed to enhance the educational process. Supportiveness is not just to provide help but also includes interactive behaviors such as offering comfort and exchanging material resources, knowledge, and information (Sarros & Sarros, 1992). A considerable number of studies in social support and well-being have verified that social support can relieve an entity of pressure, maintain mental health, and increase prosperity at work (Holt-Lunstad et al., 2010; Toker, 2011). Social support is significantly related to well-being and a significant factor for predicting well-being and teaching effectiveness. Hsu and Tsai (2013) mentioned that suitable and proper social support from supervisors, peers, and families could enhance teaching effectiveness.

#### ICT Competency

Regarding the critical role of Information and Communication Technologies (ICT) in the global digitalization context (Vitanova et al., 2015), both teaching and learning effectiveness could be improved significantly. In almost all countries of the Asia Pacific region, teacher ICT enhancement programs are accessible for teachers across educational types and levels (Sahito & Vaisanen, 2017). Toward sustainable education, several European countries recommended the utilization of ICT embedment within future teacher development plans (Usun, 2009). The digital transformation process is beyond the single purpose of using ICT applications as tools but extended to the renovation of teacher roles and pedagogies toward a constructive learning environment (Valcke et al., 2007). As a result, there are also changes in the requirements, structures, and approaches of teacher continuous professional development programs (Riviou & Sotiriou, 2017).

Several factors determine teachers' ICT competencies, such as teachers' attitudes toward ICT, ICT experience and skills, self-efficacy, and perceived usefulness of ICT (Hernández-Ramos et al., 2014). The individual accountability, evaluation, career development, and formal qualifications of the teachers also play a vital role in enhancing the ICT skills that can create and maintain the link between ICT, the curriculum, teachers' needs, and professional development within a planned policies framework (Valcke et al., 2007). In short, literature has pointed out that teachers' attitudes toward the new technologies, their perceived framework guidelines, self-efficacy, and experience are crucial determinants of the ICT integration in the teaching–learning process (Valcke et al., 2007). Therefore, the overall effectiveness and efficiency of the educational activities are improved.

Considering the novel impact of the COVID-19 on education, there is a necessity of understanding factors that affect teaching effectiveness, especially under the pressure of a sudden digital transformation due to school suspension. Correspondingly, this research measures the impacts of teachers’ perspectives during COVID-19 and teachers’ ICT competencies on teaching effectiveness through the following hypotheses:

Hypothesis *1b:* Stress feeling of COVID-19 has a negative impact on online teaching effectiveness.

Hypothesis *2b:* Teacher perceived supports have a positive impact on online teaching effectiveness.

Hypothesis *3b:* School readiness toward online learning has a positive impact on online teaching effectiveness.

Hypothesis *4b:* Newly absorbed knowledge and skills during the pandemic period have a positive impact on online teaching effectiveness.

## Research Framework and Research Method

### Conceptual Framework

**Figure 1.**
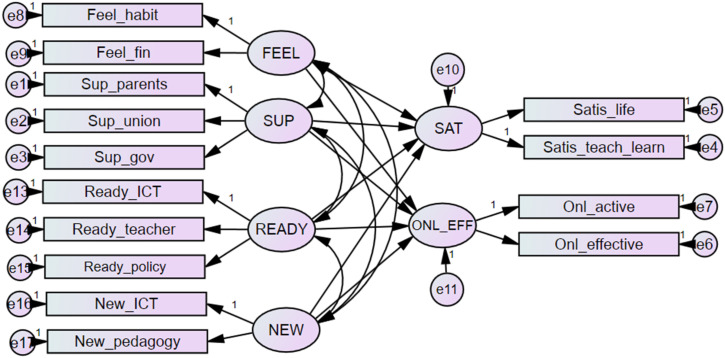
Conceptual framework of the influences of perceived supportiveness, stress feeling, school readiness, and new knowledge absorption on teachers’ satisfaction and online teaching effectiveness.

Consider teachers as the main subject of this study, this research investigated how teachers’ satisfaction and online teaching effectiveness during COVID-19 varied due to diversity in their perceptions about the pandemic’s impact. In particular, we examined teacher satisfaction (SAT) and online teaching effectiveness (ONL_EFF) during COVID-19 as the primary outcomes. Indistinct, SAT included teachers’ satisfaction on the supportiveness they received toward (i) their daily living (Satis_life) and (ii) online teaching and learning activity (Satis_teach_learn). Online teaching effectiveness was constructed by (i) teachers’ perceived teaching effectiveness (Onl_effective) and (ii) student activeness and engagement (Onl_active). Referred from the literature review, we constructed the antecedents as follows. First, teachers’ perceived perspective regarding COVID-19 (FEEL) included teachers’ feeling that there are significant changes in their living habits and financial status. The second construct is the support (SUP), which teachers receive from the Government, teacher union, and the parent association. Third, teacher readiness for online teaching (READY) is an accumulation of preparation in ICT infrastructure (Ready_ICT), school policies (Ready_policy), and teacher competency (Ready_teacher). The final construct is a function of the newly acquired technological and pedagogical knowledge (NEW). All of those above items are teachers’ self-report and were measured by a Likert scale of five (1= totally disagree, 2 = disagree, 3 = neither disagree nor agree, 4 = agree, and 5 = totally agree). Figure 1 demonstrates the overall structural equation model (SEM) to examine the relationship of four factors on teacher satisfaction and online teaching effectiveness under the adverse situation.

### Data Collection

The research protocol has been approved by EdLab Asia Educational Research and Development Centre’s IRB (No.200402). Researchers collected these data from April 6, 2020 to April 11, 2020, 2 months since the first date of school closure due to COVID-19 in Vietnam. Initially, a preliminary test was conducted with the involvement of thirty K-12 teachers and four principals in Hanoi to validate the measurements. After revising the questionnaire, we spread the survey URL across the Facebook groups of Microsoft Innovative Educator and Vietnam Innovative Education Forum–the most popular online community of Vietnamese teachers, with 38,600 members and 14,000 members, respectively. Within 1 week, there were 1005 clicks on our survey, which led to 373 potential observations. However, the final dataset includes 294 observations only, due to the exclusion of 79 respondents which violated our cross-checking questions. The final dataset has been stored in Harvard Dataverse (Hoang et al., 2020c), and the full descriptive statistic can also be found in Data in Brief (Vu et al., 2020).

## Results and Discussion

### Descriptive Statistic

Table 1 presents the demographic of 294 respondents. The majority of respondents are female (83.3%), come from public schools (65.0%), and teachers mostly have bachelor’s degrees (61.6%). Regarding teaching experience, nearly half of the teachers (41.8%) have more than ten years of teaching experience. Regarding teaching subjects, the distribution is quite equal, namely, 29.6% in sciences-related, 23.8% in social sciences-related, 19.4% in foreign languages, and 27.2% in other subjects. Many teachers are teaching in primary school (34.0%), while lower, upper, and post-secondary school teachers account for 21.4%, 22.4%, and 19.0%, respectively.

**Table 1.** Demographic and Basic Information of Respondents.

Characteristic	Respondents	
	Frequency (*n* = 294)	%
***Gender***		
Male	46	15.6
Female	245	83.3
Prefer not to disclosure	3	1.0
***Teaching experience***		
From 1 to 3 years	64	21.8
From 3 to 5 years	48	16.3
From 5 to 10 years	59	20.1
More than 10 years	123	41.8
***Teaching qualification***		
Diploma	13	4.4
BA	181	61.6
MA	89	30.3
Doctor	11	3.7
***School type***		
Public school	191	65.0
Private (normal)	49	16.7
Private (bilingual/international)	37	12.6
Continuing education center	13	4.4
Other	4	1.4
***Subject***		
Sciences-related	87	29.6
Social sciences-related	70	23.8
Foreign language	57	19.4
Others	80	27.2
***Grade level***		
Pre-K	9	3.1
Primary	100	34.0
Lower secondary	63	21.4
Upper secondary	66	22.4
Post-secondary	56	19.0

### Measurement Model

#### Measurement Validation

We used SPSS 20 to conduct confirmatory factor analysis (CFA) and AMOS to run structural equation modeling (SEM). Initially, we assessed to find out whether our model has acceptable goodness of fit, as suggested by a two-step approach (Anderson & Gerbing, 1988). The results for the goodness of fit are presented in Table 2. As can be seen from the table, the Chi-square of the model is 73.596, the degree of freedom is 55, and the adjusted Chi-square over the degree of freedom is 1.338 (smaller than 3) (Mantel, 1963). Moreover, the goodness of fit (GFI) is 0.966 (>0.95), and the adjusted goodness of fit (AGFI) is 0.935 (>0.90) which indicates a well-fitting model (Hooper et al., 2008). Finally, the model reports normed-fit index (NFI) of 0.957 (>0.95), root-mean-square error (RMSEA) of 0.034 (<0.08), and comparative fit index (CFI) of 0.988 (>0.95) (Hooper et al., 2008). All in all, all indices suggest a good fitting model.

**Table 2.** Results of Multiple Fit Indices.

Index	Result	Acceptable level
Chi-square	*73.596*	-
Degree of freedom	*55*	-
Chi-square/Degree of freedom	1.338	<5
GFI	*.966*	>0.9
AGFI	*.935*	>0.8
NFI	*.957*	>0.9
RMSEA	*.034*	<0.08
CFI	*.988*	>0.9

Table 3 demonstrates the outputs of factor loading for CFA at *p* < 0.001. With 294 observations in our study, a factor loading value of 0.50 is required for each item (Hair et al., 1998). From Table 3, all elements in their related constructs have high enough factor loading values. As a result, four factors (perceived support, stress feeling, school readiness, and new knowledge absorption), as well as teacher satisfaction and online teaching effectiveness, are measured by their reliable indicators.

**Table 3.** Results of Factor Loading for Confirmatory Factor Analysis.

Items	Factor loading
FEELING (FEEL)	
Feel_fin	0.588
Feel_habit	0.856
SUPPORT (SUP)	
Sup_parents	0.735
Sup_union	0.831
Sup_gov	0.89
NEW KNOWLEDGE (NEW)	
New_ICT	0.856
New_pedagogy	0.767
SCHOOL READINESS (READY)	
Ready_ICT	0.771
Ready_policy	0.878
Ready_teacher	0.863
ONLINE TEACHING EFFECTIVENESS (ONL_EFF)	
Onl_active	0.954
Onl_effective	0.701
TEACHER SATISFACTION (SAT)	
Satis_teach_learn	0.697
Satis_life	0.886

Finally, Table 4 shows the results of the convergent and discriminant validity test. All of the measurement constructs have composite reliability (CR) bigger or equal to 0.7, average variance extracted (AVE) bigger than 0.5 (Byrne, 2013), and maximum shared variance (MSV) smaller than AVE (Fornell & Larcker, 1981). The maximum reliability (MaxR(H)) was also examined. Subsequently, the model’s discriminant validity is constructed (Hancock & Mueller, 2001). Besides, outputs of factor correlations confirm the negative influence of teacher satisfaction, online teaching effectiveness, and teachers’ feeling.

**Table 4.** Convergent and Discriminant Validity.

Construct	CR	AVE	MSV	MaxR(H)	Factor correlation					
					SAT	SUP	FEEL	READY	NEW	ONL
SAT	0.775	0.635	0.194	0.821	**0.797**					
SUP	0.861	0.674	0.194	0.878	0.441	**0.821**				
FEEL	0.7	0.539	0.116	0.766	−0.340	−0.087	**0.734**			
READY	0.876	0.703	0.170	0.886	0.401	0.241	0.034	**0.839**		
NEW	0.795	0.661	0.170	0.807	0.122	0.118	0.127	0.412	**0.813**	
ONL_EFF	0.821	0.701	0.088	0.917	0.163	0.085	−0.297	0.128	0.232	**0.837**

#### Structural Model

Table 5 illustrates the outputs for SEM. As can be seen from the coefficients, the perceived support and school readiness have positive relationships with teacher satisfaction. In particular, the more support teachers received from both inside and outside schools, the more appreciation they have. Among various supports, the support from the government is the most crucial to teachers, with the estimated correlation of 0.79; support from parents association weights 0.54 only (the detailed interactions between constructs and their related factors can be seen in Supplementary Appendix 1). Moreover, if the schools are more ready for the transformation (online learning), the teachers would feel more satisfied. The readiness related to policies and teachers’ capabilities contributed most to general school readiness according to teachers’ views (0.77 and 0.74, respectively), while ICT infrastructure accounted for a smaller part (0.59). However, these factors (perceived support and school readiness) have no impact on teachers’ perception of online teaching effectiveness.

**Table 5.** Results of Structural Equation Model.

	Coefficient	*p*	Hypothesis
*Dependent variable: SAT*			
*SUP*	0.334	***	H2a supported
*FEEL*	−0.32	***	H1a supported
*READY*	0.341	***	H3a supported
*NEW*	−0.022	0.752	H4a not supported
R^2^	39%		
*Dependent variable: ONL*			
*SUP*	0.016	0.809	H2b not supported
*FEEL*	−0.33	***	H1b supported
*READY*	0.029	0.691	H3b not supported
*NEW*	0.26	***	H4b supported
R2	16.3%		

Chi-square = 73.223; degree of freedom = 54; Cmin/df = 1.356; goodness of fit (GFI) = 0.966; adjusted goodness of fit (AGFI) = 0.934; normed fit index (NFI) = 0.957; root mean square error of approximation (RMSEA) = 0.035; The Tucker–Lewis coefficient (TLI) = 0.988 and comparative fit index (CFI) = 0.988

In addition, stress feeling in teachers has a negative relationship with both teachers’ satisfaction and teachers’ perception of online learning, and the two influences are quite similar in weight (−0.32 and −0.33). In other words, when teachers feel they have to change daily habits or their financial plan is threatened due to COVID-19, they tend to have lower satisfaction, and they consider online teaching is less effective. Between the change in daily habit and financial plan, teachers are affected more by daily habit changes, with the estimated correlation of 0.73 compared to 0.34 of the financial threat.

The final remarkable relationship is between newly absorbed knowledge and skills and teachers' perception of online learning. This positive relationship shows that the more teachers acquire new pedagogy and ICT knowledge and skills, the more students' engagement and online effectiveness they perceive. For the respondents, new ICT knowledge and expertise is slightly more critical than pedagogy- related knowledge and skills (a difference of 0.14). Additionally, teachers' perception of online learning focuses a lot on students' active engagement (0.91) compared to the effectiveness of teaching and learning (0.49).

## Conclusion

Teacher satisfaction is a crucial element toward teaching commitment and teaching effectiveness, which contribute to sustainable education development. The main goal of this study is to explore teachers’ satisfaction and teaching effectiveness during the sudden digital transformation of teaching and learning due to COVID-19. On the one hand, this study contributes to broadening the literature on educational operation during crises. On the other hand, it highlights essential aspects, which educational leaders can consider enhancing the stable efficiency of teaching and learning activities during such a chaotic situation of COVID-19. In short, the research team figured out the significant influences of teacher’s perceived support, stress, and readiness over teacher satisfaction. To promote online teaching effectiveness, school leaders should pay attention to teachers’ stress and anxiety, as well as enhancing online teaching competencies.

First, the final dataset of 294 observations has built up a shred of convincing evidence to confirm the influence of stress over teachers’ satisfaction and online teaching effectiveness. In particular, sudden changes in daily routine and teaching habits due to school closure, as well as the anxiety regarding the current and potential decrease in regular income, had significant negative impacts on both the teachers’ satisfaction and the online teaching effectiveness. This result is associated with what was explored long ago that variation in psychology may lead to the alteration of teaching effectiveness (Barr, 1952) and that teachers are dissatisfied with such extrinsic factors as contextual or societal effects as declared in previous scholars (Day, 2013; Dörnyei & Ushioda, 2013).

Second, the perceived support from students’ parents, from the unions or relevant authorities and the readiness of the schools, which influenced the most on the teachers’ satisfaction, made no impact on the online teaching effectiveness. By this finding, the physical and mental extrinsic support seems to induce adverse effects on teachers during the COVID-19 pandemic compared to their regular teaching lifetime. Whereas under the usual educational conditions, the significant dissatisfiers with teachers are noted to be parents, policymakers, business leaders, or politicians (Hargreaves & Fullan, 1998); they play vital roles in raising the teachers’ satisfaction in adversity.

Third, among tested factors, the newly absorbed knowledge or skills was the only mediator which can help to enhance the online teaching effectiveness. However, incorporating new pedagogical and technological skills did not improve teachers’ satisfaction during the pandemic. This finding is a notable contribution to current works of literature on teacher continuous professional development, in which learning new knowledge and skills is a critical component of teacher self-autonomy toward life-long learning and long-term career satisfaction (Little, 1995; Pearson & Moomaw, 2005).

Inclosing this article, we would like to mention considerable limitations of the work, which can be fulfilled by future studies. First, with the focus on online teaching effectiveness, this research did not include the sample of teachers in mountainous and island areas, whose ICT infrastructure and Internet access are limited. Second, the findings of this research mostly relied on teachers’ self-report within a small group of teachers. Thus, future open database should be constructed to increase the diversity of this research topic (Vuong, 2020). As a result, a part of the data might be exaggerated as a consequence of teachers’ stress due to COVID-19, while the other part might be flattened. Last but not least, the situation of COVID-19 was well handled by the Vietnamese government, so the stress of Vietnamese teachers might not be as acute as their colleagues in other countries, especially the territories with high levels of COVID-19 spreading. Thus, a comparative study will be beneficial to portrait the picture of teacher satisfaction during a pandemic.

## Supplemental Material

sj-pdf-1-jex-10.1177_00220574211039483 – Supplemental Material for Factor Associated With Teacher Satisfaction and Online Teaching Effectiveness Under Adversity Situations: A Case of Vietnamese Teachers During COVID-19 Click here for additional data file. Supplemental Material, sj-pdf-1-jex-10.1177_00220574211039483 for Factor Associated With Teacher Satisfaction and Online Teaching Effectiveness Under Adversity Situations: A Case of Vietnamese Teachers During COVID-19 by Phuong-Tam Pham, Thanh-Thao Thi Phan, Yen-Chi Nguyen and Anh-Duc Hoang in Journal of Education
