# Supplementary figures and images for: Factor Associated With Teacher Satisfaction and Online Teaching Effectiveness Under Adversity Situations: A Case of Vietnamese Teachers During COVID-19
Source: J Educ (Boston). 2021 Nov 6;203(3):605–15. doi: 10.1177/00220574211039483 (PMC10311373; doi:10.1177/00220574211039483)

## Appendix

### Appendix 1. The results of Structural Equation Model

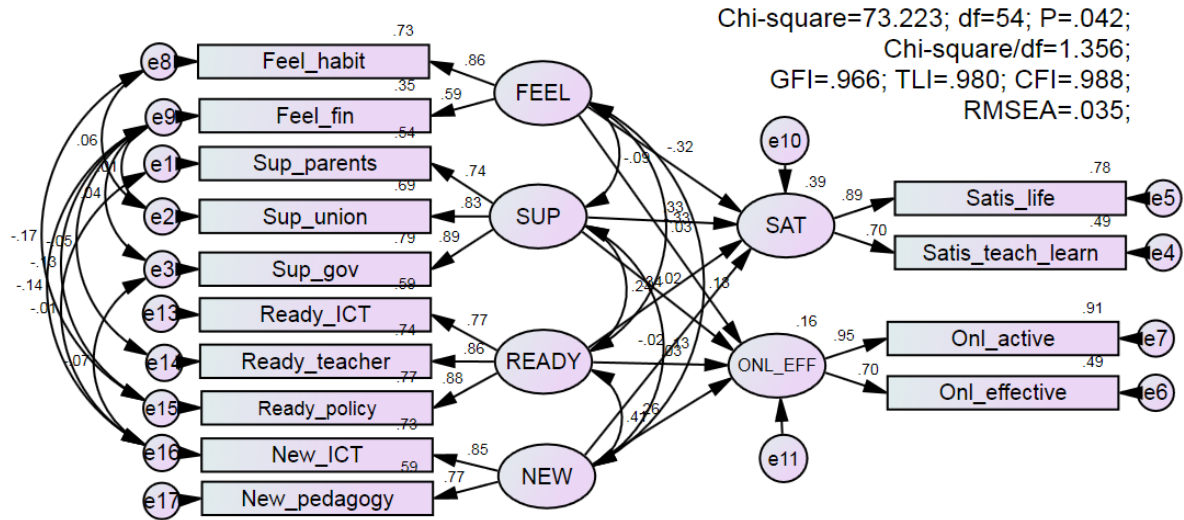

Supplement: sj-pdf-1-jex-10.1177_00220574211039483 – Supplemental Material for Factor Associated With Teacher Satisfaction and Online Teaching Effectiveness Under Adversity Situations: A Case of Vietnamese Teachers During COVID-19 [file sj-pdf-1-jex-10.1177_00220574211039483.pdf]
